# Elevated preoptic brain activity in zebrafish *glial glycine transporter* mutants is linked to lethargy-like behaviors and delayed emergence from anesthesia

**DOI:** 10.1038/s41598-021-82342-w

**Published:** 2021-02-04

**Authors:** Michael J. Venincasa, Owen Randlett, Sureni H. Sumathipala, Richard Bindernagel, Matthew J. Stark, Qing Yan, Steven A. Sloan, Elena Buglo, Qing Cheng Meng, Florian Engert, Stephan Züchner, Max B. Kelz, Sheyum Syed, Julia E. Dallman

**Affiliations:** 1 Department of Biology, University of Miami, 1301 Memorial Drive, Coral Gables, FL 33146 USA; 2 Department of Molecular and Cellular Biology, Harvard University, Cambridge, MA 02138 USA; 3 John P. Hussman Institute for Human Genomics, University of Miami, Miami, FL 33101 USA; 4 Dr. John T. MacDonald Foundation Department of Human Genetics, University of Miami, Miami, FL 33136 USA; 5 Departments of Anesthesiology and Critical Care, Perelman School of Medicine, University of Pennsylvania, Philadelphia, PA 19104 USA; 6 Pharmacology, Perelman School of Medicine, University of Pennsylvania, Philadelphia, PA 19104 USA; 7 Neuroscience, Perelman School of Medicine, University of Pennsylvania, Philadelphia, PA 19104 USA; 8 Institute for Translational Medicine and Therapeutics, Perelman School of Medicine, University of Pennsylvania, Philadelphia, PA 19104 USA; 9 Department of Physics, University of Miami, Coral Gables, FL 33146 USA; 10 Present Address: Univ Lyon, Université Claude Bernard Lyon 1, CNRS UMR 5310, INSERM U 1217, Institut NeuroMyoGène, 69008 Lyon, France; 11 Present Address: Department of Human Genetics, Emory University School of Medicine, Atlanta, GA 30322 USA

**Keywords:** Neurological disorders, Genetics, Neuroscience

## Abstract

Delayed emergence from anesthesia was previously reported in a case study of a child with Glycine Encephalopathy. To investigate the neural basis of this delayed emergence, we developed a zebrafish glial glycine transporter (*glyt1 − / −*) mutant model. We compared locomotor behaviors; dose–response curves for tricaine, ketamine, and 2,6-diisopropylphenol (propofol); time to emergence from these anesthetics; and time to emergence from propofol after craniotomy in *glyt1−/−* mutants and their siblings. To identify differentially active brain regions in *glyt1−/−* mutants, we used pERK immunohistochemistry as a proxy for brain-wide neuronal activity. We show that *glyt1−/−* mutants initiated normal bouts of movement less frequently indicating lethargy-like behaviors. Despite similar anesthesia dose–response curves, *glyt1−/−* mutants took over twice as long as their siblings to emerge from ketamine or propofol, mimicking findings from the human case study. Reducing glycine levels rescued timely emergence in *glyt1−/−* mutants, pointing to a causal role for elevated glycine. Brain-wide pERK staining showed elevated activity in hypnotic brain regions in *glyt1−/−* mutants under baseline conditions and a delay in sensorimotor integration during emergence from anesthesia. Our study links elevated activity in preoptic brain regions and reduced sensorimotor integration to lethargy-like behaviors and delayed emergence from propofol in *glyt1−/−* mutants.

## Introduction

Delayed emergence from anesthesia was reported in a case study of a girl with Glycine Encephalopathy (also known as non-ketotic hyperglycemia, NKH)^1^. GE is an inherited condition that causes elevated glycine^2–8^. In newborns, this elevated glycine inhibits motor circuits, suppressing both respiration and motor tone^9^. For those who survive this post-natal period, neural circuits adapt to high glycine. A few months after birth, infants have improved motor tone and breathe without a ventilator but continue to struggle with seizures, increased lethargy, and delays in reaching developmental milestones^9^. In this case study, a girl with GE was anesthetized for a surgical procedure, but after surgery, exhibited unexpected, delayed emergence from anesthesia^1^. Here we model delayed emergence from anesthesia in *glyt1−/−* mutant zebrafish larvae and identify differentially active brain regions in *glyt1−/− *mutants and their siblings.

Emergence from anesthesia is known to be promoted by conserved vertebrate brain arousal pathways including hypocretin, dopamine, acetylcholine, and noradrenaline^10–16^. These pathways have been best studied in rodents, where glycine has been shown to regulate hypocretin-releasing neurons and cholinergic neurons in the basal forebrain^17–19^. Both hypocretin and basal forebrain cholinergic neurons express glycine receptors^17,19^ and they are also both innervated by ascending glycinergic brainstem neurons^17,19^. Moreover, electrophysiological recordings show that application of glycine inhibits action potentials in hypocretin neurons^18^ and that glycinergic inhibitory post-synaptic potentials are present in cholinergic neurons of the basal forebrain^19^. These data support the idea that high glycine in *glyt1−/−* mutants could inhibit arousal pathways and thereby delay emergence from anesthesia.

Our zebrafish model of GE harbors a homozygous mutation in the glial glycine transporter gene (*glyt1*^*te301*^;^20,21^) and is phenocopied by raising zebrafish in a Glyt1 blocker (N[3-(4′-fluorophenyl)-3-(4′-phenylphenoxy)-propyl]sarcosine) indicating that this point mutation limits transporter function^20^. In humans, mutations in *GLYT1* have recently been shown to cause a form of GE^7,8^. In this form of GE, glycine is mildly elevated in the nervous system^7^, while in other forms caused by mutations in glycine cleavage system genes^3,4^, glycine is dramatically elevated throughout the body. Nonetheless there are overlapping symptoms between these forms of GE such as breathing difficulties and reduced motor tone^7^.

Here, we show that prior to anesthesia *glyt1−/−* mutant zebrafish have normal bouts of movement but initiate these bouts less frequently. *glyt1−/−* mutants take more than twice as long as their siblings to emerge from two structurally distinct general anesthetics: ketamine and 2,6-diisopropylphenol (the active ingredient of propofol). Whole-brain activity mapping during baseline conditions shows that preoptic brain regions are more active in *glyt1−/−* mutants while widespread sensorimotor activation is suppressed. Reducing brain glycine in *glyt1−/−* mutants restores timely emergence from anesthesia, pointing to elevated glycine as causal. Cumulatively, our results support a model whereby high glycine in *glyt1−/−* mutants promotes activity in preoptic brain regions and interferes with sensorimotor integration to produce lethargy-like behaviors and delayed emergence from anesthesia.

## Methods

### Animals

 Wild type (Tubingen Longfin and Brian’s Wild Type) and *glyt1*^*te301*^^20^ heterozygous adult *Danio rerio* strains were maintained on a 14-h light, 10-h dark circadian cycle at 28.5 °C in recirculating Aquatic Habitats (Apopka, FL) aquaria. The *glyt1 shocked *^*te301*^ genotype was determined using PCR followed by restriction enzyme digest as in Mongeon 2008^20^. All larvae were raised in ‘system water’ (reverse osmosis water conditioned with salts and bicarbonate that houses adults). Larvae at five and six days of age have not completed sexual maturation and therefore sex is not indicated. All procedures were reviewed and approved by the University of Miami Institutional Animal Care and Use Committee and are described in protocols 13-212 and 16-217 entitled ‘A zebrafish model of delayed emergence from anesthesia in patients with glycine encephalopathy.’ The University of Miami has an Animal Welfare Assurance on file with the Office of Laboratory Animal Welfare (OLAW), National Institutes of Health (assurance number: A-3377-01). It has had continuous accreditation by the Association for Assessment and Accreditation of Laboratory Animal Care (AALAC) since 1960. All experiments were performed in accordance with the relevant guidelines and regulations of these agencies.

### Kinematic analyses

 High-speed videos were captured at 1000 frames per second (fps) with a frame resolution of 512 × 512 pixels using a FastCAM 1024 PCI high-speed camera (Photron, San Diego, CA). Larvae were filmed in a custom plexiglass enclosure with the camera and associated Fujinon 1:1.4/25 mm CF25HA1 lens (Fujifilm North America Corporation, Valhalla NY) mounted 8.25 cm below a 35 mm Petri dish containing eight to twelve larval zebrafish. Backlighting mounted above the larvae was provided by a CS420 constant current source LED array (Advanced Illumination, Rochester, VT). To evoke escape responses, we used brief vibration stimuli provided by a Mini-shaker 4810 (Brüel and Kjær, Denmark) attached to a titanium rod and platform that held the Petri dish of zebrafish larvae. The vibration was controlled by a Dell Quad Duo computer via a data acquisition card (PCI-6221; National Instruments, Austin, TX), connection block (BNC-2110; National Instruments, Austin, TX) and a Grass S48 Stimulator (Astro-Med, Inc., W. Warwick, RI).

### Four-hour recordings in lanes

For four-hour daytime recordings, five-day-old larvae were placed in custom-made lanes. Lanes were fabricated by milling a 0.5 × 7.25 × 10 cm (h × w × l) piece of plexiglass with 15 0.5 × 0.3 × 6.5 cm (h × w × l) wells. A Logitech HD 720p webcam was placed below the wells to avoid reflections associated with the meniscus.

### Code

Videos were analyzed with custom-written MATLAB (MathWorks Inc., MA) scripts: https://github.com/sheyums/ChiyuanLI-Fish-Code. Larval positions in each image were determined by the ‘background subtraction’ method. Position was defined by larva’s center-of-mass, calculated from larval average × and y pixel locations. To compensate for slowly changing environment due to water evaporation and mechanical fluctuations in the tank, the algorithm automatically updated the background every ~ 1000 frames.

### Anesthesia

Stock solutions of 100 µM of 2,6-Diisopropylphenol (Propofol; SAFC supply, St. Louis, MO) and 0.4% buffered tricaine methanesulfonate salt (Sigma, St Louis, MO) were diluted in system water. Fresh stock solutions were prepared at the beginning of each experiment. Ketamine was purchased from the Division of Veterinary Services at the University of Miami as a 10 mg/mL stock solution in water (Vedco, St. Joseph, MO) and also diluted in system water.

Propofol solutions were made in two ways, with and without sonication. *Without sonication*, 1 µL of neat propofol was added to 1 mL of system water followed by 30 s of vortexing. This mixture was further diluted into 50 mL and vortexed again for 30 s. This method was used for data in Figs. 3, 4, 5, and 6. *With sonication*, a calibrated, drawn out glass pipet was used to measure and transfer neat propofol to a concentration of ~ 100 µM (1 µL/50 mL system water in a glass bottle) before vortexing for 30 s and sonicating (Cole-Parmer Sonogen 60,626) for five minutes^22^. Sonication reduces the amount of bath propofol needed to anesthetize animals by ten-fold. This method was used for data in Figs. 2 and Supplementary Fig. 2 and 3. To compare how much propofol reached the brain tissue using each method, we carried out HPLC on dissected brains exposed to bath propofol without or with sonication (Supplementary Fig. 1).

#### Dose/Response

 Twelve-well plates with basket inserts were used. Five larvae (6 days post-fertilization) were placed in each basket/well containing 1.5 ml of system water (Fig. 2A). The well plate was then placed in the Noldus DanioVision chamber for 1 h so larvae could dark adapt. Following dark adaptation, a visual-motor response assay (VMR) coupled with a vibrational stimulus was used to assess larval state. Larvae were exposed to 30 s of lights-on (12% intensity in Noldus, 1200 lx) and 20 s of lights-off conditions followed by a tap (at intensity 5) and left in the dark for another 10 s. This cycle was repeated for five minutes. Next, larvae were transferred to increasing concentrations of anesthetic, the stimulation protocol repeated, and the proportion of larvae responding was recorded. The proportion responding in the 5 s before and after tap/light responses during 4th and 5th VMR cycles at each dose was subsequently fitted with a sigmoidal dose–response curve using Prism (GraphPad) as in^22^. For tricaine, the concentrations tested were 76.5 µM, 95.7 µM, 114.8 µM, 153.1 µM, 191.4 µM, 306.2 µM, 421 µM, and 765.4 µM. For ketamine, the concentrations tested were 50 µM, 100 µM, 500 µM, 1 mM, 5 mM, and 10 mM. For propofol the concentrations tested were 0.05 µM, 1 µM, 2 µM, 4 µM, 6 µM, 8 µM, and 10 µM.

#### Emergence from anesthesia

 After washout of each anesthetic, larvae were tested at five-minute intervals for recovery of movement in response to vibration. The larval vibration-elicited escape response is similar to assays used in frog tadpoles^23^ and the righting reflex in mice used to assess emergence from anesthesia^24^. Mixed genotype larvae from a *glyt1* heterozygous cross were exposed to anesthesia, allowed to recover, and then euthanized with tricaine before genotyping.

#### Craniotomy

*glyt1−/−* mutant larvae were anesthetized in tricaine, placed dorsal side up on a slanted plate made with 1% agarose in system water, and surgically manipulated to introduce a hole in the roof of the brain using sharpened tungsten needles as in^20^. Larvae were then placed in modified Hanks solution: in mM 140 NaCl, 0.1 Na_2_PO_4,_ 3 KCl, 0.2 K_2_PO_4_, 3 mM D-Glucose so as not to osmotically shock exposed brain ventricles, a protocol previously used to normalize glycine levels in *glyt1−/−* mutants^20^.

### MAP-mapping with pERK/tERK staining compared across treatments and genotypes.

MAP-mapping was carried out as in Randlett et al. 2015^25^. 16–18 larvae per batch were fixed and stained with pERK and tERK antibodies (Cell Signaling Tech) followed by fluorescent secondary antibodies. Samples were imaged dorsal side up on an upright confocal with voxel size 0.08 × 0.08 × 2 µm using a 20 × water immersion objective. To capture the entire brain, two regions were stitched together. Using t-ERK staining, brain z-stacks were warped to a reference brain using CMTK. After warping, stacks were down-sampled to resolutions of 300, 679, and 80 sections in x, y, and z planes respectively and smoothed with a 2D Gaussian filter using the macro “PrepareStacksForMAPmapping.ijm” in Fiji. To compare staining across treatments, pERK values were divided by tERK values on a per voxel basis to normalize for individual variability in staining.

### Experimental design

The majority of experiments were carried out blind to genotype on five and six-day-old larvae. No statistical power calculation was conducted prior to the study and sample sizes were based on the available data. Homozygous *glyt1−/−* mutants were compared to their siblings (*glyt1* ± and +/+). To enrich for *glyt1−/−* mutants and thus reduce the total number of animals used, embryos were dechorionated at 28–30 h post-fertilization and sorted for their ability to move (*glyt1−/−* mutants are paralytic at this stage), then remixed with a similar number of siblings, and reared to days five and six for experiments. At least three batches of larvae were used per experiment to control for batch effects.

### Statistical analysis

**Figure 1:**
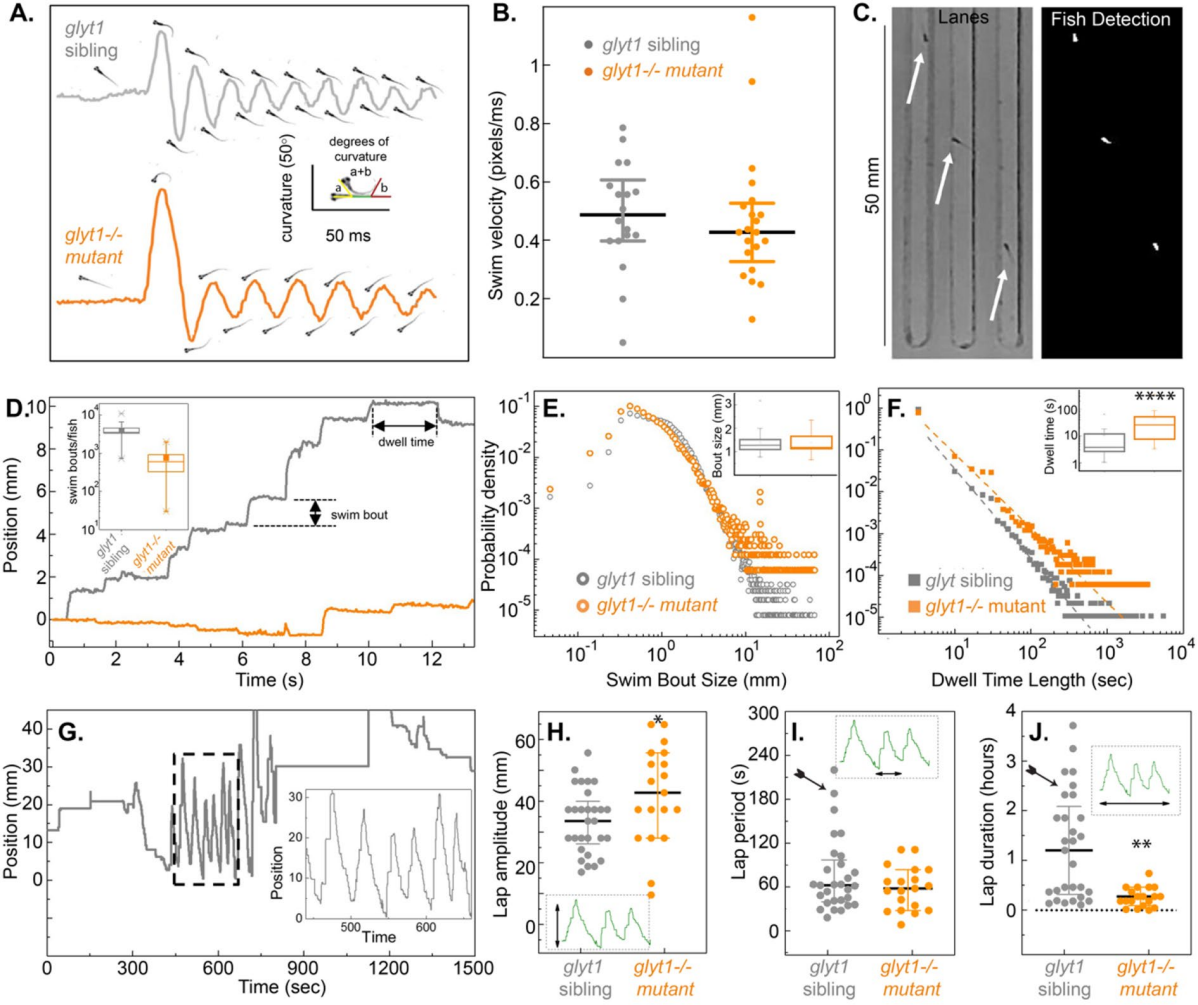
*glyt1−/−* mutants show normal bouts of locomotion with reduced bout frequency. (**A**) Body curvature is graphed over time for representative wild type sibling (grey) and *glyt1−/−* mutant (orange). Images of each larva at times of peak curvature are shown. (**B**) Median swim velocity with interquartile ranges are plotted for wild type siblings (n = 18) and *glyt1−/−* mutants (n = 21). (**C**) We designed custom lanes for measuring basal locomotor activity. Single larvae (white arrows; left panel) are placed in each lane and recorded for four hours. Binary image (white, right panel) shows larvae used for quantitative image analysis in Matlab. (**D**) Raw data of fish position versus time are plotted for a representative *glyt1* sibling and *glyt1−/−* mutant. Periods of inactivity are interrupted by periodic bouts of swimming. Inset box plots show that *glyt1−/−* mutant fish are less active and more variable in activity than their wild type siblings. The probability densities (y-axis label in E also serves for F) with insets showing median and interquartile interval for (**E**) swim bout distance and (**F**) dwell times for *glyt1−/−* mutants (n = 16,427 bouts from 24 larvae) and *glyt1* siblings (n = 125,784 bouts from 30 larvae). Mann Whitney rank tests show bout size is not different (*p* = 0.99) while dwell times are significantly longer in *glyt1−/−* mutants *p* = 1.18 × 10^(− 5). Asterisks indicate *p*-value: *p < 0.05, **p < 0.001, ***p < 0.001, ****p < 0.0001. (**G**) Position of an individual fish over fifteen minutes is plotted to show lap behavior. While *glyt1−/−* mutants have larger lap amplitudes than their siblings (*p* = 0.03) (**H**), *glyt1−/−* mutants do not exhibit prolonged durations for single lap behaviors (**I**). Clustered lap behaviors were less frequent in *glyt1−/−* mutants than in their siblings (*p* = 0.0003; see arrows).

Prism (GraphPad) and Matlab were used for graphing and statistical analyses. Datasets were analyzed for normal distributions in Prism and their majority were not normally distributed. Therefore, nonparametric analyses were used. For basal behavior (Fig. 1) and emergence from anesthesia experiments (Table 1 and Figs. 3B, 4B and 7), non-parametric Kruskal Wallis ANOVAs are followed by Dunn’s Multiple Comparisons to calculate *p*-values and 2way-ANOVAs are followed by Sidak’s multiple comparisons. For datasets in Figs. 1, 3, and 4 medians ± interquartile range are reported with *p*-values based on non-parametric Mann–Whitney test (abbreviated MW). For the pERK/tERK MAP mapping approach in Figs. 5 and 6, voxel intensities between two groups of 16–18 larvae were compared with the Mann–Whitney U-statistic Z score as in^25^. A false discovery rate (FDR)-based method was used to set the significance threshold. The color intensity (0–65,535) assigned to the voxel is proportional to the difference between the median values. For the projections, intensity is scaled linearly with saturation at 60% of the maximum pixel intensity. This analysis was carried out in Matlab using the function “MakeTheMAPMap.m.” Regions that exceed the threshold for significance are indicated in either green (greater relative activity in treatment 1) or purple (greater relative activity in treatment 2).

**Table 1:** *glyt1−/−* mutant larvae take longer to emerge from propofol and ketamine but not tricaine.

Experiment	ANOVA (Kruskal Wallis)		Dunn’s multiple comparisons					
Anesthesia (minutes)			*glyt1* + */* + vs. *glyt1* ±		*glyt1* + */* + vs. *glyt1−/−*		*glyt1* + */ − *vs. *glyt1−/−*	
	H statistic	*p*	Rank	*p*	Rank	*p*	Rank	*p*
Propofol (30)	28.5	< 0.0001	− 4.6	> 0.99 ns	− 30.5	< 0.0001	− 25.8	< 0.0001
Propofol (120)	32.1	< 0.0001	5.1	> 0.99 ns	− 28.8	0.0001	− 34.0	< 0.0001
Ketamine (45)	14.8	0.0006	1.9	> 0.99 ns	− 12.8	0.01	− 14.7	0.0004
Ketamine (120)	27.7	< 0.0001	4.2	> 0.99 ns	− 16.4	0.007	− 20.6	< 0.0001
MS222 (60)	4.1	0.13 ns	1.6	> 0.99 ns	− 7.5	0.61 ns	− 9.1	0.14 ns
MS222 (120)	1.2	0.55 ns	− 1.8	> 0.99 ns	− 4.2	0.85 ns	− 2.4	> 0.99 ns
*No Surgery* Propofol (30)	20.6	< 0.0001	7.1	0.47	− 13.9	0.03	− 21.0	< 0.0001
*Surgery* Propofol (30)	1.2	0.56 ns	3.5	0.96 ns	1.4	> 0.99 ns	− 2.1	> 0.99 ns

Times to emergence from different anesthetics are compared across *glyt1* genotypes. Shown are Kruskal Wallis ANOVA H-statistic and *p*-value followed by Dunn’s multiple comparisons rank and *p*-value.

We did not exclude any animals from our analyses.

## Results

### ***glyt1−/−mutants show normal bouts of locomotion but reduced bout frequency***

Previous work had shown that *glyt1−/−* mutant larvae are initially paralyzed by elevated glycine but recover coordinated locomotion by four days post fertilization^20,26^. Locomotion is central to assessing anesthesia in this model, therefore, here we carried out a more thorough assessment of swim bout coordination, frequency, and stamina in five-day-old *glyt1−/−* mutants and their siblings.

We first used high-speed recordings to compare discrete bouts of locomotion. As shown previously in Mongeon et al. (2008), both *glyt1−/−* mutants and siblings responded to vibration with a pronounced C-bend away from the stimulus followed by alternating flexions along the body axis (Fig. 1A). There was no difference between *gylt1* mutants and their siblings in swim velocity (Fig. 1B; *p* = 0.35; MW). These results show that five-day-old *glyt1−/−* mutants have normal bouts of locomotion.

We next made long-term (4-h) recordings at reduced temporal resolution sufficient to capture the beginning and end of each bout of locomotion. We placed larvae in lanes (Fig. 1C) that allowed them to both swim long distances relative to their body length (Fig. 1D) and to easily turn and swim in the opposite direction. From these recordings, we extracted quantitative measurements and constructed probability densities of both distance traveled per swim bout (Fig. 1E) and dwell times (the time in between bouts; Fig. 1F). We found that while swim bouts were similar in size between *glyt1−/−* mutants and their sibling (Fig. 1E; *p* = 0.999; MW), dwell times between movements were longer in *glyt1−/−* mutants, indicating that mutants initiate swim bouts less frequently (Fig. 1F; *p* = 1.18 × 10^−5^; MW).

In these arenas, zebrafish larvae also showed lap swimming during which larvae swam back and forth along the lane by producing many sequential bouts of locomotion with only short intervening rests (Fig. 1G; hatched box). This lap behavior had characteristic lengths (Fig. 1H), periods (Fig. 1I), and duration (Fig. 1J). Although *glyt1−/−* mutant laps were slightly larger in amplitude than their siblings (*p* = 0.03, MW), individual siblings showed the ability to swim for longer periods than any of the *glyt1−/−* mutants (Fig. 1I; arrow). *glyt1* siblings also maintained swimming for longer periods of time than *glyt1−/−* mutants (Fig. 1J; *p* = 0.0003; MW; arrow). In sum, these long-term recordings show that despite normal *glyt1−/−* mutant bouts of locomotion, *glyt1−/−* mutants initiate these bouts less frequently and exhibit shorter sustained periods of movement. We define reduced frequency of movement as ‘lethargy-like behaviors’ similar to those described in other animal models^27^ and in individuals with GE^28^.

### ***glyt1−/− mutants have similar propofol dose–response curves to aversive tap stimuli***

**Figure 2:**
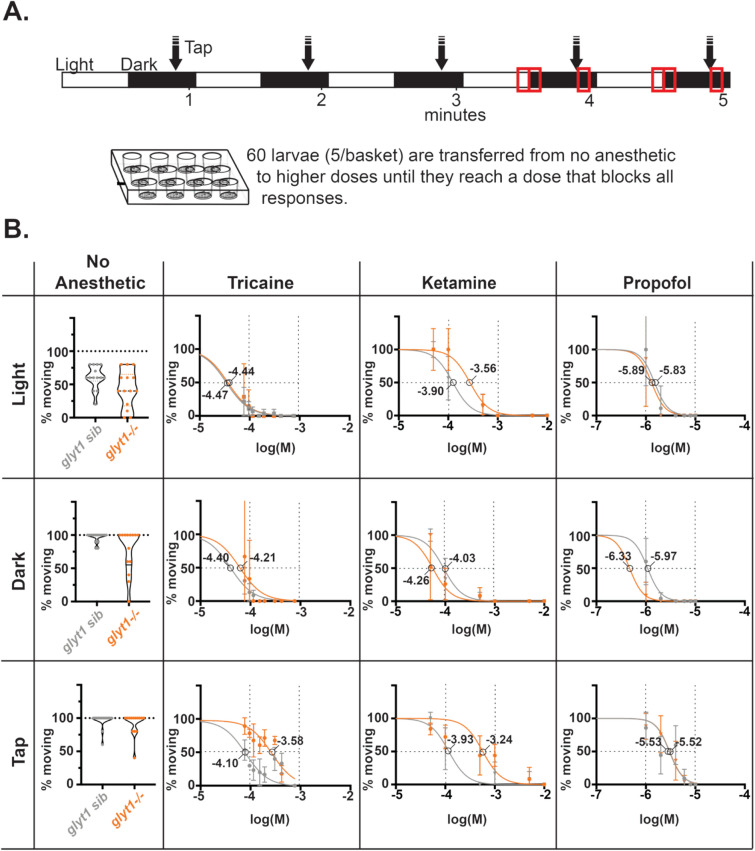
*glyt1* siblings and *glyt1−/−* mutants have similar tricaine, ketamine, and propofol dose–response curves. (**A**) Our experimental approach is diagrammed. Larvae in 16-well plates, with five larvae per basket insert, were exposed to alternating thirty second intervals of light and dark, with a tap delivered after 20 s in dark; this sequence of stimuli was repeated five times for a total of five minutes. Measurements of larval swimming were made at minutes four and five (red boxes). Larvae were then exposed to increasingly higher concentrations of anesthetic by transferring baskets until all stopped moving in response to tap. (**B**) The percentage of *glyt1* sibling (gray) and *glyt1−/−* mutant (orange) moving are plotted for three behavioral endpoints: swimming in light (top row), swimming in response to a dark transition (middle row), and swimming in response to a tap (bottom row). Percentage responding are shown for each batch in the absence of anesthetic (left-most column; n = 21 batches of five larvae for both *glyt1−/−* mutants and their siblings). To the right, normalized mean percentages moving are plotted against log M concentrations of Tricaine (second column; n = 3 batches of five larvae for both *glyt1−/−* mutants and their siblings), Ketamine (third column; n = 5 batches of five larvae for both *glyt1−/−* mutants and their siblings), and propofol (fourth column; n = 14 batches of five larvae for both *glyt1−/−* mutants and their siblings). Dose–response data were fitted with nonlinear four parameter curves constraining Hill slope in Prism. Horizontal dashed lines show 50% response and EC50 for each curve is indicated on the graphs.

We compared anesthesia induction in *glyt1−/−* mutants and their siblings with three anesthetics that act through different pathways: tricaine, a non-specific Na channel blocker and the most commonly used anesthetic in zebrafish^29^, ketamine, a general anesthetic and NMDA receptor blocker^30^, and propofol, a general anesthetic and potentiator of GABA and glycine receptors. We also directly compared three behavioral endpoints for dose–response curves: loss of spontaneous swimming behavior, loss of response to transitions from light to darkness (visual motor response, VMR), and loss of response to tap/vibrational stimuli (Fig. 2A). In the absence of anesthesia, larvae are more active in response to dark transitions than in the light (*glyt1* sib *p* = 0.0013; *glyt1−/−* mutant *p* = 0.0229) and more active in response to tap than in the light (*glyt1* sib *p* = 0.0005; *glyt1−/−* mutant *p* = 0.0002; Fig. 2B).

The proportions of larvae responsive at different anesthetic doses were first normalized and then fit with polynomial four parameter curves in Prism. By testing these three behavioral endpoints sequentially, we observed that zebrafish tend to stop swimming and lose their VMR response at lower anesthetic concentrations than what is required to suppress the tap response (Fig. 2B; Supplementary Fig. 2; Supplementary Table 1). As such, we consider loss of swimming/VMR response similar to sedation as an endpoint while loss of the tap response, a more aversive stimulus, as more similar to anesthesia. While *glyt1−/−* mutants tended to become sedated at lower anesthetic doses than their siblings, they show similar or reduced anesthetic sensitivity in the tap assay. Doses of ketamine required to suppress the tap response also reduced larval heart rate (data not shown), therefore, for subsequent ketamine experiments, we used doses that were sedative but not fully anesthetic.

### ***Time to emergence from propofol and ketamine is delayed in glyt1−/− mutants***

**Figure 3:**
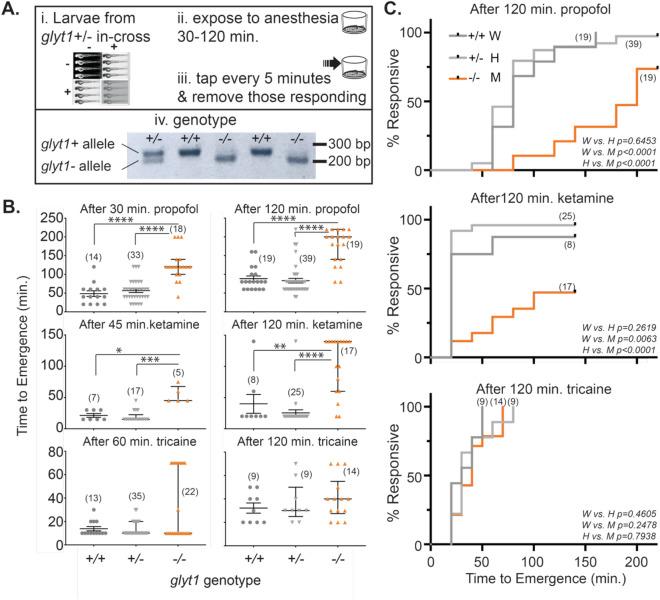
*glyt1−/−* mutant zebrafish larvae show delayed emergence from anesthesia. (**A**) Experimental workflow is indicated. i-Progeny from *glyt1* ± adult in-cross were raised for five days and ii-transiently exposed to one of three anesthetics:10 µM propofol, ketamine (20 mM 45 min. or 10 mM 120 min.) and buffered 765.4 µM tricaine. iii-After removal of anesthetic, time of first response to vibration (stimuli were delivered at five-minute intervals) was recorded and iv-individual genotypes were subsequently determined using a PCR/restriction enzyme-based assay. A representative, cropped image of the genotyping gel with each lane corresponding to a larvae is shown. (**B**) Scatter plots of individual emergence times are overlaid with median and interquartile range. Kruskal Wallis ANOVAs followed by Dunn’s multiple comparisons were conducted for each anesthetic and incubation time. Asterisks indicate *p*-value: *p < 0.05, **p < 0.001, ***p < 0.001, ****p < 0.0001. Compared to their siblings, *glyt1−/−* mutants took more than twice as long to emerge from both ketamine and propofol. By contrast, *glyt1−/−* mutants and their siblings took similar amounts of time to emerge from tricaine. (**C**) Kaplan–Meier plots of the proportions of responsive fish against time-post-anesthesia for the same *glyt1* + */* + , *glyt1* + */-*, and *glyt1−/−* larvae presented in B are shown post-120-min exposures to propofol (top), ketamine (middle), and tricaine (bottom). *p*-values are calculated using log rank tests.

To determine the relationship between *glyt1* genotype and time to emergence from anesthesia, we compared time to regain tap response after exposure to the three anesthetics in *glyt1* + */* + , *glyt1* ± , and *glyt1−/−* larvae (Fig. 3A; Supplementary Fig. 3). While there was no difference between *glyt1* genotypes in time to emergence from 765.4 µM tricaine, *glyt1−/−* mutants took significantly longer than their siblings to emerge from either 10 mM ketamine or 10 µM propofol (Fig. 3B; Table 1). As would be predicted, longer exposure to anesthetics was associated with correspondingly longer times to emergence (Fig. 3B; propofol 30 vs. 120 min. exposure, *p* < 0.0001; ketamine 45 vs. 120 min. exposure, *p* = 0.0004; tricaine 60 vs. 120 min. exposure, *p* < 0.0001; MW). Moreover, failure-time-analyses showed that *glyt1−/−* mutants took more than twice as long as their siblings to emerge from anesthesia after two-hour exposures to propofol and ketamine but not to tricaine (Fig. 3C; Log rank test).

### ***Craniotomy accelerates emergence from propofol in glyt1−/− mutants***

**Figure 4:**
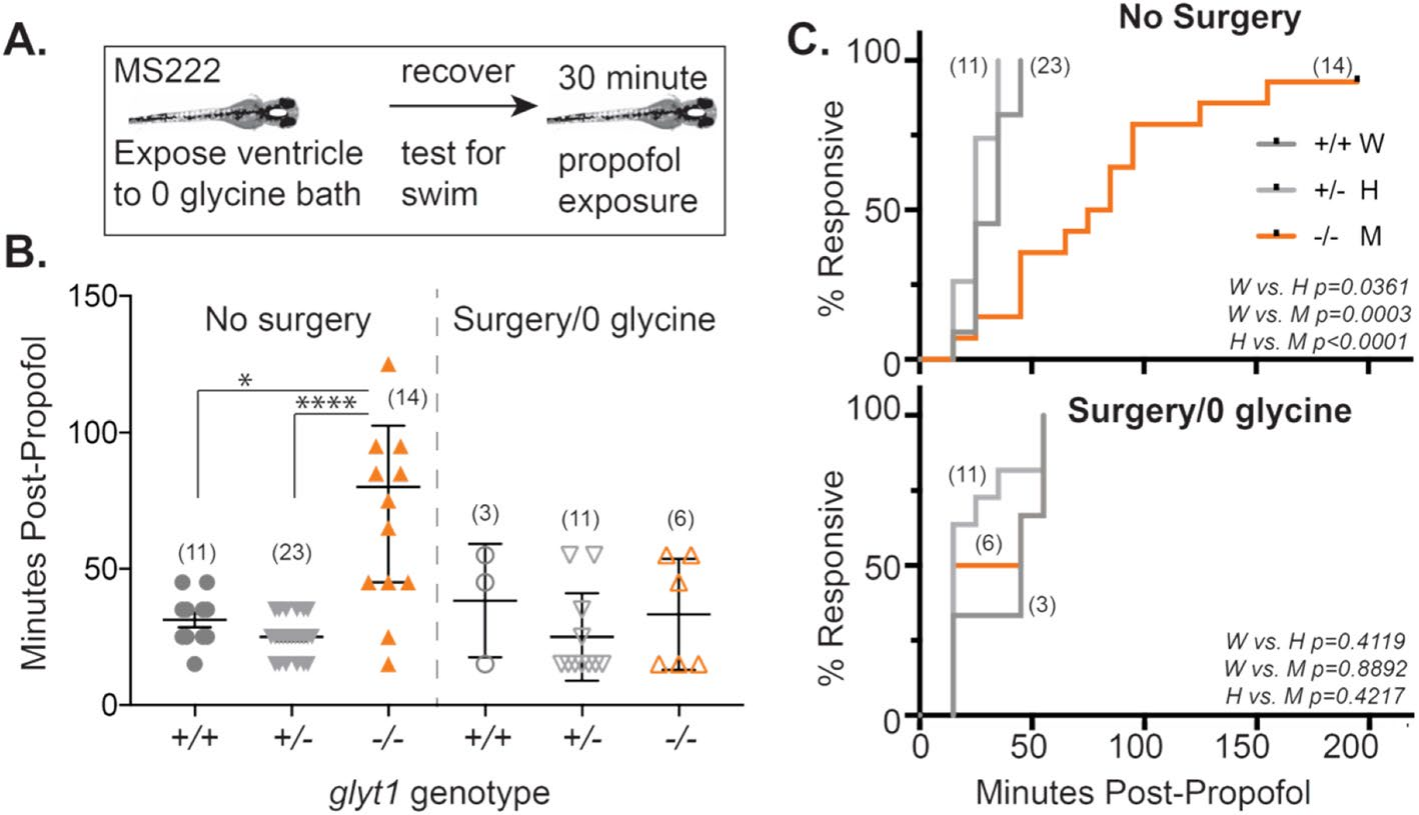
Reducing glycine accelerates emergence from propofol in *glyt1−/−* mutants. (**A**) To test the role of glycine in delayed emergence from anesthesia, we anesthetized five-day-old *glyt1* sibling (+ /- light gray; + / + dark gray) and mutant (*− / − *orange) larvae with tricaine, surgically exposed brain ventricles to a 0-glycine bath solution, tested larvae for intact swimming behavior and then anesthetized them with propofol for thirty minutes and measured their time to recovery. Numbers in each experimental group are indicated in parentheses on the plots in B and C. (**B**) Scatter plots of times to emergence post-propofol are plotted for larvae that were not surgically manipulated (left) and larvae with surgically exposed brain ventricles (right). Results were analyzed with a two-way ANOVA (treatment and genotype) followed by Wilcoxon/Kruskal Wallis test of significance. Asterisks indicate *p*-value: *p < 0.05, **p < 0.001, ***p < 0.001, ****p < 0.0001. *glyt1−/−* mutants emerged significantly more slowly than wild type siblings and *glyt1−/−* mutants with surgically exposed brain ventricles. (**C**) Kaplan–Meier plots of the proportions of responsive fish against time-post-propofol for the same *glyt1* + */* + , *glyt1* + */-*, and *glyt1−/−* larvae presented in B are shown for No Surgery (top) and Surgery/0 glycine (bottom). *p*-values are calculated using log rank tests.

To test whether elevated glycine in *glyt1−/−* mutants could explain delayed emergence, we surgically exposed brain ventricles to a bath solution by incising the skin over the fourth ventricle (craniotomy)^20^, thus equilibrating brain and bath glycine levels (Fig. 4A). This procedure was previously shown to reduce brain glycine levels sufficiently to restore movement to pre-recovery, two-day-old *glyt1−/−* embryos that would otherwise be paralyzed by elevated glycine^20^. Here, we used tricaine to anesthetize five-day-old *glyt1−/−* mutant and their sibling larvae for craniotomy surgery and let them recover swimming behaviors prior to exposing them to 10 µM propofol for 30 min and measuring time to emergence (Fig. 4B; Table 1). With surgery, *glyt1−/−* mutants had emergence times similar to their siblings and emerged significantly faster than *glyt1−/−* mutants without surgery*.* These results indicate that delayed emergence from propofol in *glyt1−/−* mutants is caused by a humoral factor, most likely glycine, in the cerebral spinal fluid of *glyt1−/−* mutants.

### ***In both glyt1−/− mutant and their siblings, propofol inhibits brain-wide activity***

**Figure 5:**
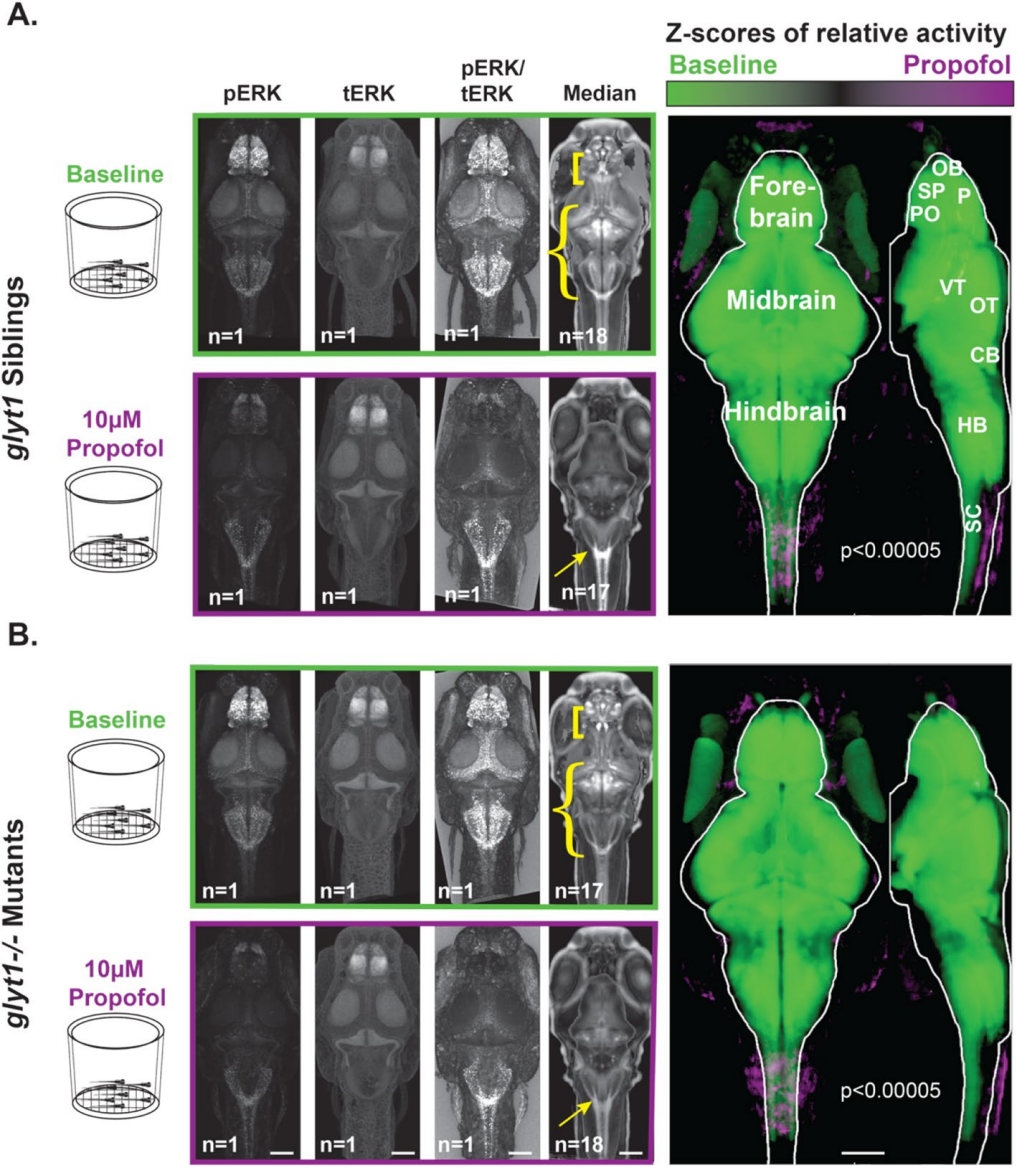
Brain-wide activity in both *glyt1−/−* mutants and wild type siblings is significantly reduced by exposure to propofol. Four batches of sixteen to eighteen, six-day-old larvae were placed in baskets (diagramed to the left) to enable easy transfer of larvae from system water to 10 µM propofol solutions. Half contained *glyt1* siblings (**A**) and the other half *glyt1−/−* mutants (**B**). Larvae from each treatment were fixed and stained with pERK and tERK antibodies and z-stacks of the brain were captured on a confocal microscope. Images boxed in green correspond to baseline conditions while images boxed in purple correspond to exposure to 10 µM propofol for twenty minutes. Images show standard deviation projections of pERK, tERK, pERK/tERK, and median values. To compare activity between baseline and propofol treatments, voxels with more intense pERK/tERK at a *p* < 0.00005 threshold under baseline conditions are shown in green while voxels with more intense pERK/tERK in propofol are shown in purple for *glyt1* siblings (**A**) and *glyt1−/−* mutants in (**B**). It is clear that both *glyt1* siblings and *glyt1−/−* mutants have reduced activity at anesthetic doses of propofol as expected from their similar dose/response curves. Yellow brackets, curly brackets and arrows in median images highlight staining patterns that differ significantly between *glyt1−/−* mutants and their siblings. Under Baseline conditions, brackets point to a region encompassing the subpallium and preoptic that has more pronounced staining in the *glyt1−/−* mutants while curly brackets point to a region encompassing the optic tectum, cerebellum, and hindbrain that has more intense staining in the *glyt1* siblings. Under propofol, arrows point to the area postrema region of the hindbrain that has more pronounced staining in *glyt1* siblings. CB cerebellum, HB hindbrain, OB olfactory bulb, OT optic tectum, P pallium, PO preoptic, SC Spinal Cord, SP Sub-pallium, VT ventral tegmentum. Scale bars = 100 µm.

**Figure 6:**
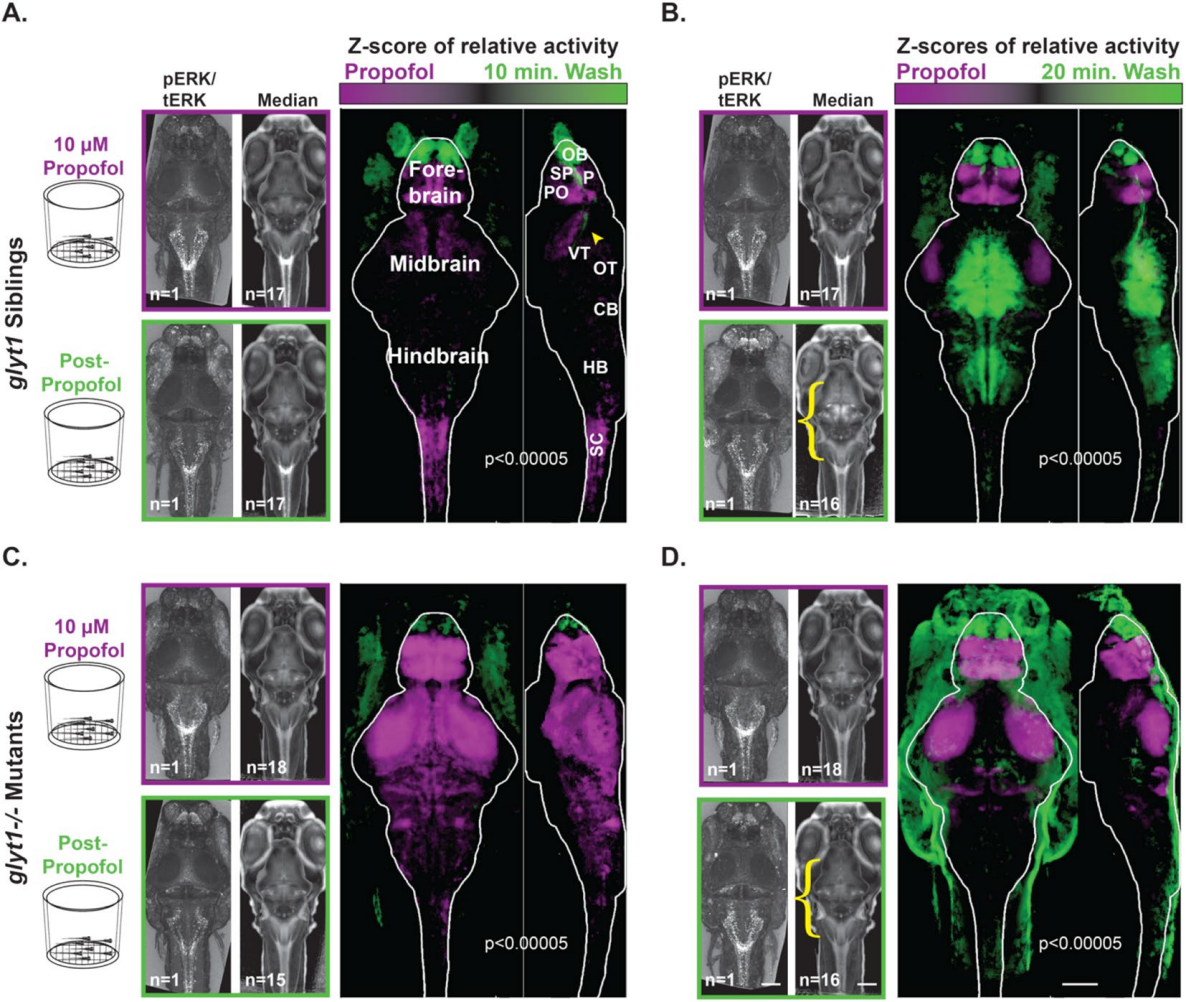
Compared to *glyt1* siblings, *glyt1−/−* mutants show delayed activation of pre-motor, and motor regions during emergence from propofol. Four batches of sixteen to eighteen, six-day-old larvae were placed in baskets (diagramed to the left) to enable easy transfer of animals from 10 µM propofol to system water solutions. Half contained *glyt1* siblings after (**A**) ten minutes and (**B**) twenty minutes in system water. The other half contained *glyt1−/−* mutants after (**C**) ten minutes and (**D**) twenty minutes in system water. To compare activity between propofol and wash treatments, voxels with more intense pERK/tERK at a *p* < 0.00005 threshold under propofol conditions are shown in purple while voxels with more intense pERK/tERK in system water during emergence are shown in green for *glyt1* siblings (**A**, **B**) and *glyt1−/−* mutants in (**C**, **D**). *glyt1* siblings show greater activation of preoptic, subpallium, and spinal cord regions under propofol, with the olfactory bulb and a regions of the diencephalon that encompasses the arousal pathways (yellow arrowhead in **A**) being the first brain region to become more active during emergence from propofol, followed ten minutes later by the optic tectum, cerebellum and hindbrain. *glyt1−/−* mutants have greater activation of preoptic and subpallium, but also optic tectum neuropil and cerebellar neuropil under propofol. As with their *glyt1* siblings, the olfactory bulb is the first brain region to become activated but there is no activation of other sensory, pre-motor, and motor regions by the twenty-minute timepoint consistent with delays seen in *glyt1−/−* mutants recovery of locomotor behaviors after propofol. Scale bars = 100 µm.

To compare brain-wide activity in *glyt1−/−* mutants and their siblings, we used the MAP-mapping approach^25^. Twenty larvae were placed in baskets to facilitate transfers between system water, 10 µM propofol, and fixative (diagramed in Figs. 5 and 6). After fixation, larvae were stained with antibodies against phospho-ERK (pERK; Fig. 5 left-most column) and total-ERK (tERK; Fig. 5 second column). The pERK/tERK ratio (Fig. 5 third column) was used as a proxy for neuronal activity integrated over the 15 min just prior to fixation as in^25^. Median values per voxel were then calculated for different treatment conditions (Fig. 5; fourth column). To compare the relative activity between baseline and 10 µM propofol, pERK/tERK values were compared on a per voxel basis and the threshold for significance was corrected for multiple comparisons (*p* < 10^−5^). Green voxels indicate higher pERK/tERK values in the baseline condition while purple voxels indicate higher pERK/tERK values during exposure to 10 µM propofol (Fig. 5; right-most column). As expected, both *glyt1−/−* mutants and their siblings showed significantly more intense brain-wide activity under baseline conditions (green) than in 10 µM propofol (purple).

**Figure 7:**
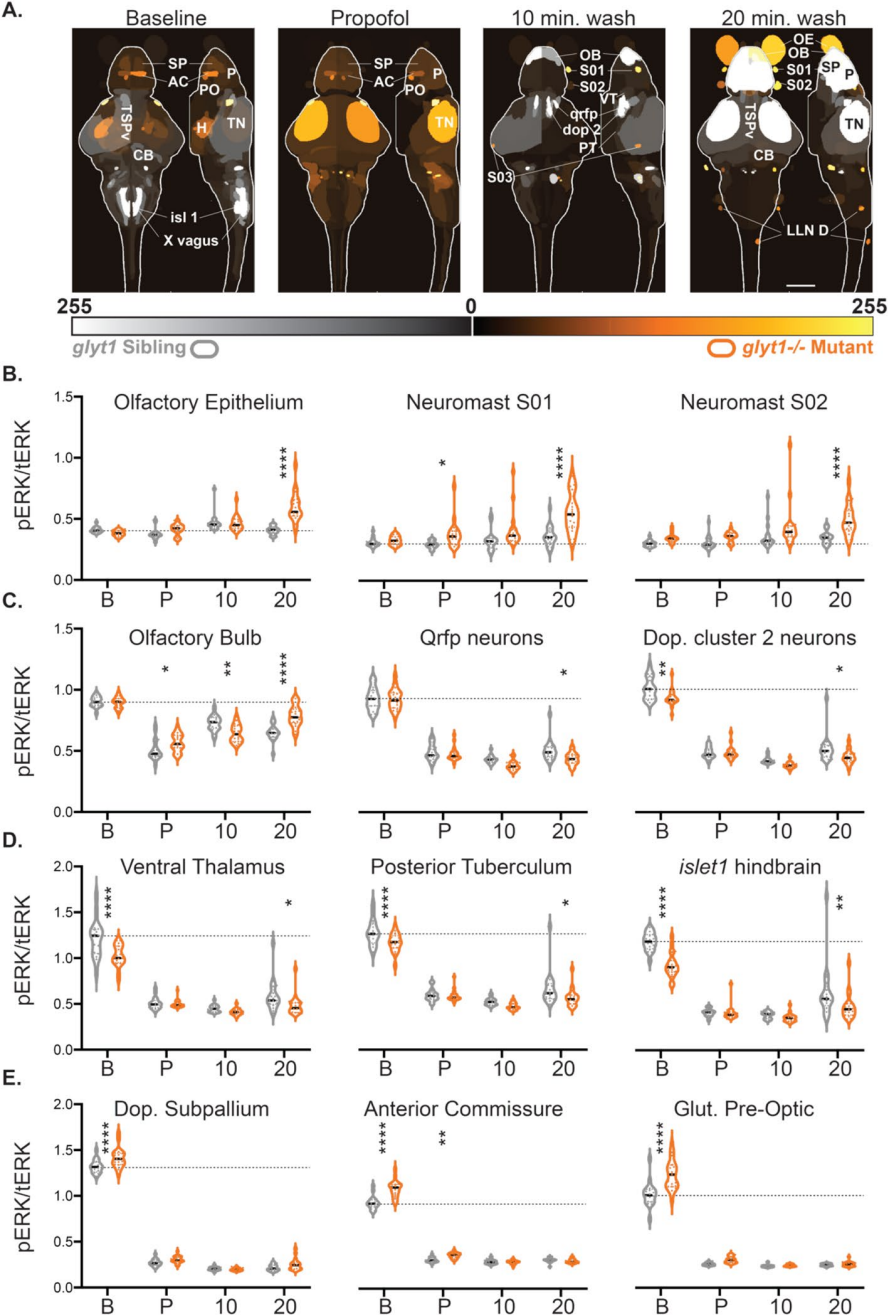
*glyt1−/−* mutants’ sensory ganglia are more active while arousal and motor circuits are less active than their siblings during recovery from propofol. (**A**) Brain regions of interest (ROIs) that are significantly different between *glyt1−/−* mutants and their siblings are shown on reference brains for Baseline , Propofol, 10 min. wash and 20 min. wash conditions with sample size indicated at the base of each image. ROIs are labeled with the corresponding anatomical brain regions; an abbreviation key can be found at the end of the figure legend. (**B**–**D**) Violin plots of pERK/tERK ratios for twelve different brain regions are shown with a dashed line indicating median pERK/tERK levels in baseline *glyt1* siblings. For each brain region, we conducted a 2-way ANOVA for genotype and condition followed by Sidak’s multiple comparisons of genotype across conditions. Asterisks indicate *p*-value: *p < 0.05, **p < 0.001, ***p < 0.001, ****p < 0.0001. (**A**) Three sensory ganglia are shown that are elevated in glyt1 mutants during recovery. (**B**) Olfactory bulb and arousal pathways are shown. (**C**) Premotor and motor regions elevated in baseline *glyt1* siblings are shown. (**D**) Hypnotic regions elevated in baseline *glyt1−/−* mutants are shown. Anatomical abbreviations: **OE** olfactory epithelium, subpallium **SP**, pallium **P**, Preoptic nucleus **PO**, Hypothalamic nucleus enriched in Qrfp-expressing neuronal cell bodies **Qrfp**, Ventral Thalamus **VT**, Dopaminergic Cluster 2 in the posterior tuberculum **Dop 2**, Tectum Stratum Periventriculare **TSPv**, Tectum Neuropil **TN**, Cerebellum **CB**, vagal motor nucleus **X**, posterior Lateral Line Neuromasts along the body **LLN D**. Scale bar = 100 µm.

To identify brain regions associated with propofol exposure and emergence from propofol, we compared activity in 10 µM propofol to activity both 10 and 20 min after propofol washout in *glyt1* siblings (Figs. 6A,B and 7) and *glyt1−/−* mutants (Figs. 6C–D and 7). During exposure to 10 µM propofol, both *glyt1−/−* mutants and their siblings showed more activity in sub-pallial (homologous to mammalian striatum/septum^31^) and preoptic areas (homologous to mammalian preoptic^32^). Moreover, sensory ganglia and the olfactory bulb were relatively resistant to anesthesia and were also the first brain regions to become activate post-propofol (Figs. 6A,D, 7). Remarkably, even though sensory ganglia were more active in *glyt1−/−* mutants than their siblings post-propofol, motor regions were less active, indicating reduced sensorimotor integration in *glyt1−/−* mutants (Fig. 7B–D). These brain regions align with mammalian literature linking activity in preoptic circuits to hypnotic states of sleep and anesthesia^33,34^, and heightened activity in sensory circuits to emergence from anesthesia^35^.

### ***glyt1−/− mutants show elevated activity in the preoptic, a hypnotic brain region***

We next compared activity between *glyt1−/−* mutants (orange) and their siblings (gray) during baseline, exposure to 10 µM propofol, and at both 10 and 20 min after washing out propofol (Figs. 7; Table 2). Under baseline conditions, *glyt1−/−* mutants had increased activity in the subpallium, preoptic hypothalamic and the broader hypothalamus; by contrast, *glyt1* siblings had increased activity in their optic tectum, cerebellum, hindbrain, and spinal cord (Fig. 7A). These patterns indicate increased activity in sensory and motor regions in *glyt1* siblings compared to *glyt1−/−* mutants even under baseline conditions. These differences can also be seen in the median images indicated by yellow brackets and arrows (Fig. 5). Under baseline conditions, preoptic and sub-pallial brain regions show stronger staining in *glyt1−/−* mutants (straight yellow brackets) while optic tectum, cerebellum, and hindbrain show stronger staining in their siblings (curlicue yellow brackets).

**Table 2:** Brainwide differences between *glyt1−/−* mutant and sibling larvae are most pronounced in baseline and 20 min. wash conditions.

Brain region	2Way ANOVA Genotype/condition			Sidak’s multiple comparisons *glyt1−/−* vs. Siblings *p*-value			
	Interact	Geno	Cond	Baseline	Propofol	10 wash	20 wash
OE	** < 0.0001**	** < 0.0001**	** < 0.0001**	0.5997 ns	0.1782 ns	0.9969 ns	** < 0.0001**
S01	**0.0025**	** < 0.0001**	** < 0.0001**	0.9793 ns	0.0375	0.0547 ns	** < 0.0001**
S02	0.0956	** < 0.0001**	** < 0.0001**	0.4619 ns	0.3674 ns	0.0538 ns	** < 0.0001**
OB	** < 0.0001**	** < 0.0001**	**0.0178**	0.9985 ns	0.0290	0.0024	** < 0.0001**
PT-Dop 2	0.0539	** < 0.0001**	** < 0.0001**	**0.0023**	> 0.9999 ns	0.2273 ns	**0.0127**
Qrfp	0.1401	** < 0.0001**	**0.0034**	> 0.9999 ns	0.9412 ns	0.1095 ns	**0.0141**
VT	**0.0059**	** < 0.0001**	** < 0.0001**	** < 0.0001**	0.9992 ns	0.8347 ns	**0.0420**
PT	0.1298	** < 0.0001**	**0.0003**	**0.0085**	0.9997 ns	0.5204 ns	**0.0265**
Isl 1	**0.0004**	** < 0.0001**	** < 0.0001**	** < 0.0001**	> 0.9999 ns	0.9587 ns	**0.0051**
SP-Dop	**0.0056**	** < 0.0001**	**0.0001**	** < 0.0001**	0.3229 ns	0.9899 ns	0.1927 ns
AC	** < 0.0001**	** < 0.0001**	** < 0.0001**	** < 0.0001**	**0.0082**	0.9971 ns	0.9715 ns
PO-Glut	** < 0.0001**	** < 0.0001**	** < 0.0001**	** < 0.0001**	0.2256 ns	0.9986 ns	0.9970 ns

For the twelve brain regions shown in Fig. 7, 2way ANOVA *p*-values for interaction, genotype, and condition are shown in columns 2–4 are followed by *p*-values for Sidak’s multiple comparisons of *glyt1−/−* mutants and siblings across the four conditions in columns 5–8.

Surprisingly, during 10 µM propofol, *glyt1−/−* mutants showed more activity overall than *glyt1* siblings (Figs. 6A,C and 7A). One exception to this pattern was that *glyt1* siblings show stronger staining in the area postrema (Fig. 6; yellow arrows), a brain region commonly associated with nausea, the most prevalent post-anesthesia symptom reported in humans^36^.

Ten minutes into emergence from propofol, *glyt1* siblings showed more activity in the olfactory bulb, and optic tectum, and in areas encompassing the diencephalon D2 dopaminergic, QRFP, and hypocretin/orexin arousal pathways, consistent with the *glyt1* siblings’ earlier recovery from propofol (Fig. 6A; Fig. 7A). By contrast, *glyt1−/−* mutants still showed increased activity in sub-pallial and preoptic regions, likely hypnotic circuits since these are the same regions that are more active in both *glyt1−/−* mutants and their siblings during propofol exposure (Fig. 6C,D).

At twenty minutes post-propofol, *glyt1−/−* mutants showed more activity in their sensory ganglia and olfactory bulb consistent with initiating their behavioral recovery (Supplemental Fig. 2; Fig. 6D; Fig. 7B-C). Unlike their siblings however, *glyt1−/−* mutants did not show the coordinate activation of arousal pathways in the diencephalon (Fig. 6 A,D). Moreover, at the 20 min after washing out propofol, *glyt1* siblings showed more activity in their optic tectum and cerebellum, consistent with the return of movement in behavioral assays (Supplemental Fig. 2; Fig. 6B). These comparisons indicate that during emergence in *glyt1* siblings, activity first increases in sensory before spreading to diencephalon brain regions encompassing QRFP, dopamine and hypocretin arousal pathways and motor-associated regions. By contrast, *glyt1−/−* mutants showed elevated activity in hypnotic preoptic brain regions under baseline conditions and, despite increased activity in sensory ganglia post-propofol, were delayed in activating motor brain regions during emergence from anesthesia.

## Discussion

Our work provides an animal model of delayed emergence from anesthesia in GE. GE is a rare genetic disorder impacting ~ 1:76,000 people (National Organization of Rare Disorders)^4,5^, making clinical studies of how to best anesthetize individuals with GE difficult if not impossible to conduct^1,37,38^. As such, our ability to model this phenomenon in the zebrafish *glyt1−/−* mutant provides a way to investigate underlying mechanisms of delayed emergence. As in people with GE who struggle with seizures, lethargy and failure in reaching developmental milestones^4,9^, the *glyt1−/−* mutation in zebrafish has negative impacts on long-term health, with the majority of *glyt1−/−* mutant zebrafish failing to survive juvenile periods of rapid growth. At the pre-feeding, larval stages studied here however, *glyt1−/−* mutants can produce normal bouts of swimming, having compensated for high glycine by down-regulating expression of glycine receptors, a known target of general anesthesia^20^; despite quantifiable lethargy-like behaviors reported herein, they have 100% survival and are impossible to sort by eye from their siblings based on either behavior or morphology^20^. Our findings reveal that larval *glyt1−/−* mutants have elevated activity in hypnotic, preoptic brain regions that likely explains both their lethargy-like behaviors and delayed emergence from anesthesia.

Zebrafish are a relatively unexplored model^39^ in which to study the pharmacogenetics of anesthesia^15,22,40,41^. As such, there is still variation in both the behavioral endpoints used to generate dose–response curves and in the reported doses required for anesthesia. For example, three recent studies each used different behavioral endpoints to construct dose–response curves: loss of the visual motor response (VMR)^40^ (increased swimming in larvae evoked by sudden decreases in light), loss of touch-induced escape swims^41^, and loss of the tap-induced response^22^. By directly comparing loss of spontaneous swimming, loss of VMR, and loss of tap response, we show that an order of magnitude higher dose of anesthetic was required for loss of the tap response compared to the other two behavioral endpoints, consistent with tap being a more aversive stimulus^25^. As such, we suggest that loss of VMR and spontaneous swims indicate sedation while loss of the tap response indicates a deeper state of anesthesia.

In regard to anesthetic dose, because zebrafish are aquatic, anesthetics are delivered directly to the bath. Less water soluble anesthetics like propofol form micelles in water^22^; because strategies to mitigate micelles vary, anesthetic doses reported in the literature also vary substantially^22,40,42,43^. Nonetheless, physiological measurements^42^, dose–response curves^22,40^, and HPLC measurements of brain propofol from this study support that effective anesthetic concentrations reflect differences in the way the solutions are made rather than individual differences in anesthetic sensitivity.

A challenge for zebrafish models of human inherited disorders is that zebrafish and human brains differ structurally^44^. As such, drawing parallels between zebrafish and human brain activity during different behavioral states requires physiological studies to establish that brain regions are functionally related in the two species^35^. In zebrafish, the ability to image activity in brain-wide neuronal ensembles has helped to address this problem. For example, in zebrafish as in mammals, arousal pathways including QRFP^45^, hypocretin/orexin^46,47^ and dopamine^48^ have been directly linked to increases in locomotion^49^. Moreover, in zebrafish as in mammals, activity galanin-producing neurons in the preoptic are associated with sleep^50^. Using MAP-mapping^25^, we show that activity in preoptic brain regions is also associated with anesthesia in both *glyt1−/−* mutants and their siblings, consistent with activity in the preoptic causing hypnotic states. In mammals, reciprocal inhibition between preoptic brain regions and arousal pathways is known to regulate transitions between sleep or anesthetized states and awake states^33,51–54^. What sets *glyt1−/−* mutants apart from their siblings is persistently elevated activity in preoptic brain regions that could suppress arousal pathways and contribute to their delayed emergence from anesthesia. Critically, these results highlight that delayed emergence of *glyt1−/−* mutant zebrafish is not due simply to a motor deficit.

We show that preoptic brain regions are not only more active during emergence from anesthesia but also under baseline conditions prior to exposure to anesthesia. Consistent with elevated activity in the preoptic, *glyt1−/−* mutant exhibited lethargy-like behaviors quantified by their reduced frequency of movement and reduced responsiveness to sensory stimuli. Another much less severe condition that, like glycine encephalopathy, causes both daytime lethargy^55^ and delayed emergence from anesthesia^56,57^ is narcolepsy. Narcolepsy is known to be caused by deficits in the hypocretin/orexin arousal pathway^58^, suggesting that the similar *glyt1−/−* mutant and narcolepsy phenotypes could reflect dysregulation of arousal pathways. Our study contributes to a growing body of literature supporting that distinct neural circuits mediate induction into and emergence from anesthesia with arousal pathways playing a critical role in emergence^16,59,60^.

In summary, our work follows up a case study by showing that we can recapitulate delayed emergence from anesthesia in a zebrafish *glyt1−/−* mutant model. We use this model to identify elevated activity in preoptic brain regions as likely explaining both their daytime lethargy-like behaviors and their delayed emergence from anesthesia. Rescuing time-to-emergence from anesthesia and brain-wide activity mapping support a model whereby elevated glycine promotes hypnotic pathways to delay transitions to active locomotor states. Future work that directly tests approaches to boost excitability in arousal pathways and/or suppress activity in hypnotic brain regions of *glyt1−/−* mutant could suggest therapeutic strategies for individuals with GE.

## Supplementary Information

Supplementary Information.
