# Supplementary material for: Elevated preoptic brain activity in zebrafish glial glycine transporter mutants is linked to lethargy-like behaviors and delayed emergence from anesthesia
Source: Sci Rep. 2021 Feb 4;11:3148. doi: 10.1038/s41598-021-82342-w (PMC7862283; doi:10.1038/s41598-021-82342-w)
Supplement: Supplementary file 1 — Supplementary Information. [file 41598_2021_82342_MOESM1_ESM.pdf]

Supplemental Information

Title Page, Scientific Reports

Article Title Elevated preoptic brain activity in zebrafish *glial glycine transporter* mutants is linked to lethargy-like behaviors and delayed emergence from anesthesia.

Author information: Michael J. Venincasa[1], Owen Randlett[2], Sureni H. Sumathipala[1], Richard Bindernagel[1], Matthew J. Stark[1], Qing Yan[1], Steven A. Sloan[1], Elena Buglo[1, 3-4], Qing Cheng Meng[5], Florian Engert[2], Stephan Züchner[3-4], Max B. Kelz[5-8], Sheyum Syed[9], and Julia E. Dallman[1*].

[1]Department of Biology; University of Miami; Coral Gables, FL, 33146; USA; [2]Department of Molecular and Cellular Biology, Harvard University, Cambridge, MA 02138, USA; [3]John P. Hussman Institute for Human Genomics; University of Miami; Miami, FL, 33101, USA. [4]Dr. John T. MacDonald Foundation Department of Human Genetics; University of Miami; Miami, FL, 33136; [5]Departments of Anesthesiology and Critical Care, [6]Pharmacology, and [7]Neuroscience and [8]Institute for Translational Medicine and Therapeutics, Perelman School of Medicine at the University of Pennsylvania, Philadelphia, PA 19104, USA. [9]Department of Physics, University of Miami, Coral Gables, FL, 33146, USA.

[*]**Corresponding author:** Julia E. Dallman; 1301 Memorial Drive, Coral Gables FL, 33146; (305) 284 3954; j.dallman@miami.edu

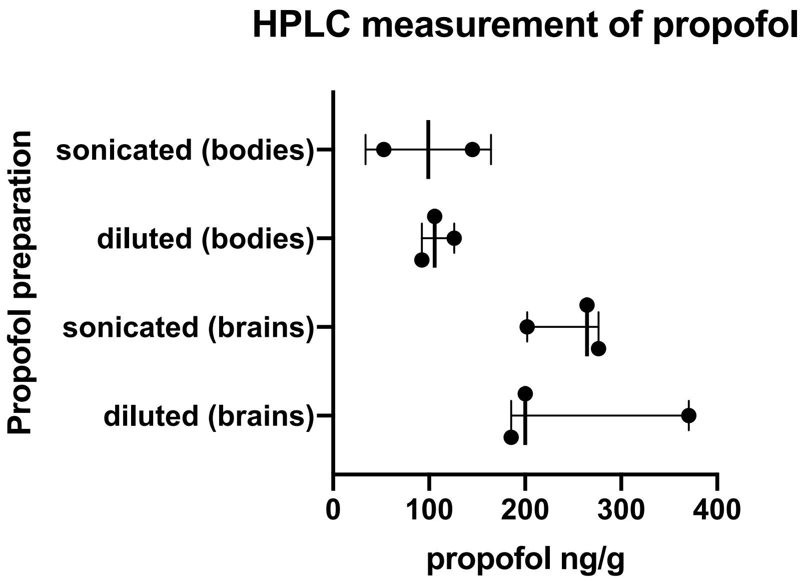

Supplementary Figure 1
We prepared neat 2,6-diisopropylphenol (propofol) in two ways, diluted and sonicated. We found that it requires more propofol prepared by dilution to anesthetize larvae, due to poor solubility. To test this interpretation, we measured propofol content in tissues, comparing the different methods of preparation and the results are graphed above. Larvae were anesthetized with propofol and frozen on dry ice before dissecting larval brains. Each sample (datapoint) contained tissue from between 16 and 20 larvae. Three independent sample replicates for both without sonication (propofol anesthetic dose bath concentration 100 μM; HPLC 252 $\pm$ 84 ng/g larval brain tissue) and with sonication (Propofol anesthetic dose bath concentration 10 μM; HPLC 277 $\pm$ 33 ng/g larval brain tissue). HPLC shows that anesthetic dose of propofol in brain tissue is the same using different ways of preparing the propofol therefore concentrations corresponding to preparation with sonication are reported throughout.

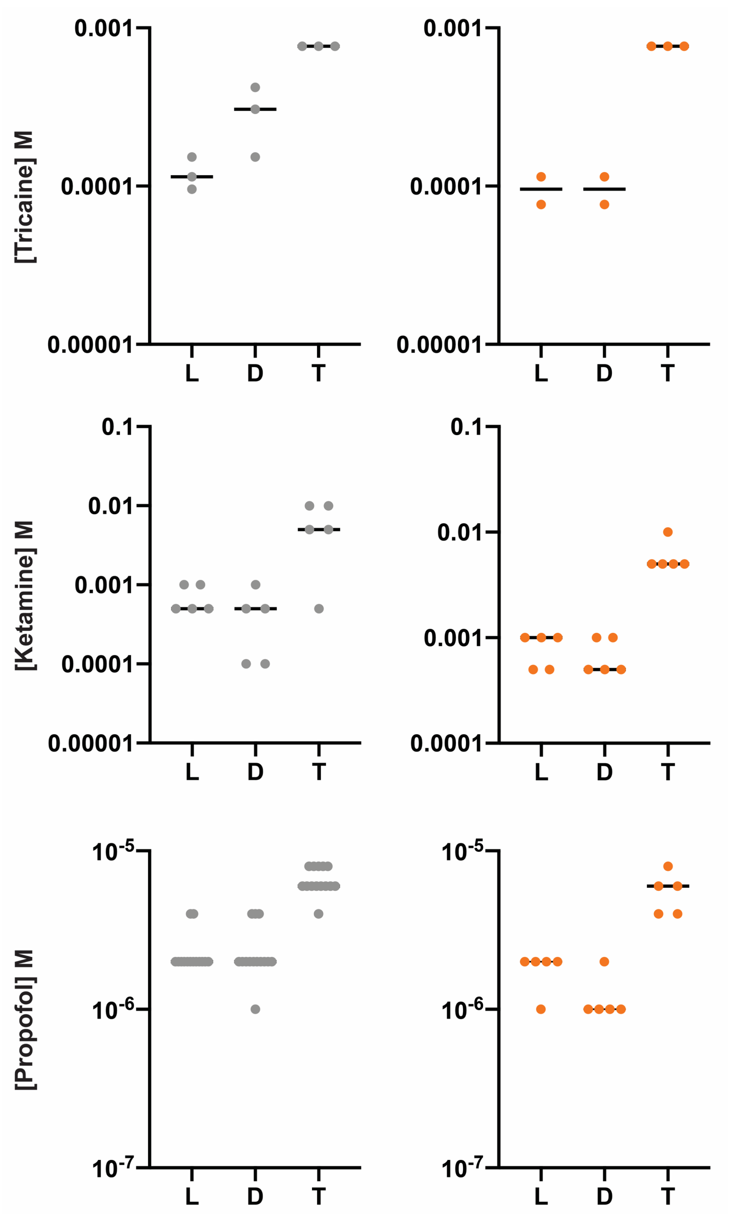

Supplementary Figure 2 Loss of swimming/response-to-stimulus occurs at an order of magnitude higher anesthetic concentrations for the tap endpoint in both *glyt1-/-* mutants and their siblings.

Each point represents a basket of five larvae and the concentration at which each group of fish stopped swimming (L=Light) or stopped responding to dark transitions (D) or tap stimuli (T) are plotted for each anesthetic and genotype.

Supplementary Table 1 Statistics for *glyt1-/-* mutant and sibling Propofol Light, Dark, and Tap endpoints.

***glyt1* Siblings**			
**ANOVA Friedman test**	**p-value**	**Number of baskets (5 fish)**	**Friedman Statistic**
	0.0001	14	26.18
**Dunns Multiple Comparisons**	**p-Value**	**Rank Sum Difference**	**Z**
**Light-Dark**	>0.9999	-1.000	0.1890
**Light-Tap**	0.0001	-21.50	4.063
**Dark-Tap**	0.0003	-20.50	3.874
***glyt1-/-* Mutants**			
**ANOVA Friedman test**	**p-value**	**Number of baskets (5 fish)**	**Friedman Statistic**
	0.0031	5	9.333
**Dunns Multiple Comparisons**	**p-Value**	**Rank Sum Difference**	**Z**
**Light-Dark**	>0.9999	3.000	0.9487
**Light-Tap**	0.1733	-6.000	1.897
**Dark-Tap**	0.0133	-9.000	2.846

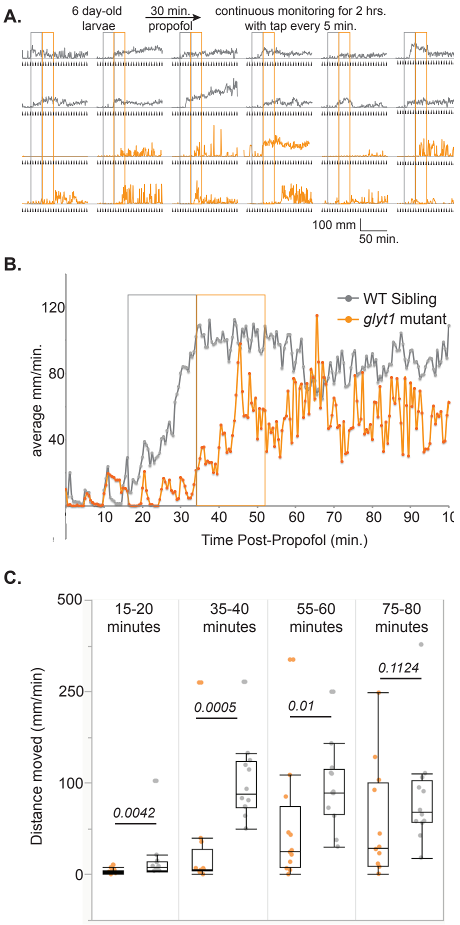

Supplementary Figure 3 Recovery of sensory/motor integration occurs in a stepwise manner and is delayed in *glyt1* mutants. *glyt1* mutant and wild type sibling larvae were exposed to vibrational stimuli at five-minute intervals after removal from propofol (30-minute exposure). Grey (WT siblings) and orange (*glyt1* mutants) boxes in A & B highlight time periods of transition from anesthetized to awake. **A**) Traces represent distance moved plotted against time for and twelve wild type sibling larvae (grey) and twelve individual *glyt1* mutants (orange). See scale bar in lower right. **B**) Mean distance traveled in mm/min. is plotted for wild type siblings (n=12; grey) and *glyt1* mutants (n=12; orange). **C**) Scatter plots compare distance moved by genotype at three times during recovery. Each point represents a larva. p-values based on a Wilcoxon test are indicated on the plot.

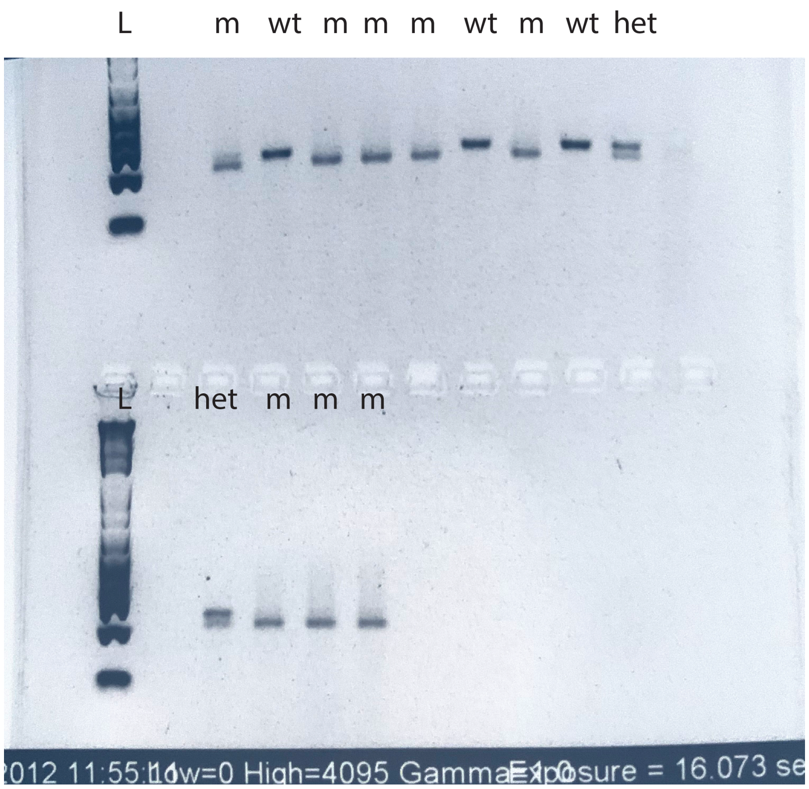

Supplementary Figure 4 An uncropped genotyping gel that was the source for the cropped image in Figure 3A is shown above. Ladders are loaded in the left-most lanes and samples are loaded to the right with genotype indicated: *glyt1-/-* m, *glyt1+/-* het, and *glyt1+/+* wt.
